# Mitigating Scatter in Mechanical Properties in AISI 410 Fabricated via Arc-Based Additive Manufacturing Process

**DOI:** 10.3390/ma13214855

**Published:** 2020-10-29

**Authors:** Sougata Roy, Benjamin Shassere, Jake Yoder, Andrzej Nycz, Mark Noakes, Badri K. Narayanan, Luke Meyer, Jonathan Paul, Niyanth Sridharan

**Affiliations:** 1 Materials Science and Technology Division, Oak Ridge National Laboratory, Oak Ridge, TN 37830, USA; 2 Mechanical Engineering Department, University of North Dakota, Grand Forks, ND 58202, USA; sougata.roy@und.edu; 3 Y-12 National Security Complex, Development Division, Consolidated Nuclear Security, LLC, Oak Ridge, TN 37830, USA; benjamin.shassere@cns.doe.gov; 4 Materials Science and Engineering Department, Virginia TechTM, Blacksburg, VA 24061, USA; jkyoder@vt.edu; 5 Oak Ridge National Laboratory, Energy and Transport Science and Technology Division, Oak Ridge, TN 37830, USA; nycza@ornl.gov (A.N.); noakesmw@ornl.gov (M.N.); meyerlt@ornl.gov (L.M.); 6 Lincoln Electric Company, Cleveland, OH 44117, USA; Badri_Narayanan@LincolnElectric.com (B.K.N.); Jonathan_Paul@LincolnElectric.com (J.P.); Niyanth_Sridharan@LincolnElectric.in (N.S.)

**Keywords:** additive manufacturing, steel, delta ferrite, microstructure, mechanical properties

## Abstract

Wire-based metal additive manufacturing utilizes the ability of additive manufacturing to fabricate complex geometries with high deposition rates (above 7 kg/h), thus finding applications in the fabrication of large-scale components, such as stamping dies. Traditionally, the workhorse materials for stamping dies have been martensitic steels. However, the complex thermal gyrations induced during additive manufacturing can cause the evolution of an inhomogeneous microstructure, which leads to a significant scatter in the mechanical properties, especially the toughness. Therefore, to understand these phenomena, arc-based additive AISI 410 samples were fabricated using robotic gas metal arc welding (GMAW) and were subjected to a detailed characterization campaign. The results show significant scatter in the tensile properties as well as Charpy V-notch impact toughness data, which was then correlated to the microstructural heterogeneity and delta (δ) ferrite formation. Post-processing (austenitizing and tempering) treatments were developed and an ~70% reduction in the scatter of tensile data and a four-times improvement in the toughness were obtained. The changes in mechanical properties were rationalized based on the microstructure evolution during additive manufacturing. Based on these, an outline to tailor the composition of “printable” steels for tooling with isotropic and uniform mechanical properties is presented and discussed.

## 1. Introduction

Additive manufacturing (AM) has gained traction over the years due to its ability to fabricate complex geometries, resulting in a significant reduction in lead times and also simplifying the supply chain [1,2,3,4]. This has resulted in a significant interest, from tool manufacturers, in using AM to fabricate near-net-shaped parts to reduce the lead times and minimize the tooling costs of machine dies. The crucial advantages are as follows: (1) shortened lead time, (2) less material waste, (3) improved functionality through incorporation of more complex features, (4) customized tooling for lower volume parts, and (5) the potential to use many materials in single parts, which can result in lowered overall costs.

While powder-based techniques have dominated the AM industry, there is a need, specifically from the tool and die manufacturers, to quickly build large components (above 10 kg), thus prompting interest in wire-arc-based additive manufacturing [5,6]. However, there are several difficulties when fabricating large scale components using wire-arc-based AM techniques. For example, Nycz et al. [7] demonstrated several key challenges while printing an excavator arm, such as print time, deposition process development, integration of the robotic path, heat management, and consistent material properties.

A range of physics-based design rules, such as overhang constraint, bead morphology, and topology optimization, need to be followed for metal big-area additive manufacturing [8,9]. Hu et al. [10] used an integrated computational and experimental approach on low carbon steel to correlate mechanical properties, microstructure and temperature history for a thin wall printed using wire arc additive manufacturing. Limited studies do exist where H13 has been used in wire-based AM and other direct energy deposition processes. Ali et al. [11] studied the wire arc additive manufacturing of tool steel using the cold metal transfer (CMT) process. They showed that, by keeping the interpass temperature above the martensite start temperature (M_s_), a homogeneous hardness level can be achieved along the height of printed samples. Ge et al. [12] deposited a crack-free sample of H13 steel. The sample dimensions were 125 mm(L) × 85 mm(W) × 47 mm(H) and were deposited on an H13 substrate. They reported a scatter in uniaxial tension and hardness, which was attributed to an inconsistent δ ferrite phase in the overlap zone. However, no study has yet demonstrated the ability to fabricate large-scale (>30 kg) H13 or other tool steel parts using wire-based AM. Therefore, there is a need to explore other printable alloy steels for tooling. The primary factor that determines the printability of steels is the carbon equivalent. To obtain crack-free prints, the selected material needs to have a low C equivalent and good machinability in the as-printed state, with the ability to be surface-hardened during post-processing. A material which fulfills these requirements is AISI 410 steel; it is a low-C-content steel that is fairly printable, and the high Cr content can make surface-hardening treatments, such as nitriding, more effective [13]. 

Due to the nature of the additive manufacturing process, there are several synergies with welding, and good weldability is a necessary condition for good printability. The following factors affect the weldability of stainless steels, and thus printability.

1. Martensitic stainless steels in general are challenging to weld due to their susceptibility to cracking [14,15]. Such steels are also susceptible to three different types of cracking which are briefly described below:
  (a) Solidification cracking: Martensitic stainless steels generally solidify as primary ferrite. Increasing the carbon content may result in solidification as austenite, which makes the material more prone to solidification cracking [16].
  (b) Reheat cracking: This occurs during post-weld heat treatments or heating of previous passes in multi-pass weldments. Impurities such as sulfur, phosphorous, boron, and copper enhance the chance of reheat cracking in the weldments [17].
  (c) Hydrogen-induced cracking: Such cracking depends on multiple factors such as composition, hydrogen content, tensile stress, and microstructure. However, due to the low C content (~0.08–0.1 wt.%) of SS410, the problem of hydrogen-induced cracking can be circumvented by application of proper preheat and interpass temperatures [18].
2. Apart from cracking, the major challenge is the scatter in mechanical properties, particularly the toughness of steel samples fabricated using additive manufacturing [19,20,21]. Previous research has attributed this to the heterogeneity in microstructure during multi-pass welding of steels. Toughness scatter in martensitic steel weldments has specifically been attributed to the presence of δ ferrite. Carrouge et al. [22] showed that reduction in the δ ferrite improved the notch toughness, even in the as-quenched condition, and upon increasing δ ferrite content from 2% to 14%, the ductile-to-brittle transition temperature (DBTT) changed from −86 °C to 46 °C. Therefore, it is important to understand the mechanisms of formation of δ ferrite and microstructure evolution during multi-pass welding of AISI 410 steel. Due to the similarities between multi-pass welding and additive manufacturing, similar microstructures can be expected [4,19,20,21].

Martensitic steels can undergo three major modes of transformation during weld metal solidification, resulting in two distinct microstructures based on welding settings and other parameters [18]: 

*Fully martensitic microstructure:* L→L + Fp (primary ferrite) (δ)→Fp (δ)→Fp (δ) + γ_Austenite_→γ_Austenite_→Martensite. The fusion zones of 11%–13% Cr steel typically solidify with a primary δ ferrite structure. The δ ferrite then transforms to γ_Austenite_ which, upon cooling, transforms completely to martensite without any δ-ferrite occurring.

*Martensite + ferrite:* If significant segregation of Cr, Mo, and C occur during solidification, a eutectic reaction occurs in which L→γ_Austenite_ + δ ferrite. Since the eutectic ferrite is rich in ferrite-stabilizing elements, it does not transform to austenite upon further cooling, leading to the formation of residual δ ferrite. However, it has been shown that this need not always be the case. For instance, Castro and de Cadenent [23] have shown that ferrite can be retained at the original ferrite dendrites due to the incomplete transformation of ferrite to austenite, similar to the formation of skeletal δ ferrite in austenitic stainless-steel welds.

In multi-pass welding, δ-ferrite can form via a solid-state mechanism in the heat-affected zone while reheating as well. Typically, stainless-steel heat-affected martensitic zones are characterized by five distinct zones:

1. Zone 1: The partially melted zone where incipient melting occurs. The temperature in this zone is above the solidus temperature of the material.
2. Zone 2: Here, the room temperature microstructure transforms completely to δ ferrite with significant grain growth, also called the coarse grain heat-affected zone.
3. Zone 3: This zone is a two-phase region which is characterized by the presence of both austenite and δ ferrite.
4. Zone 4: The peak temperature is above the upper critical temperature where the room temperature microstructure is completely austenitic
5. Zone 5: The temperature is in the inter-critical region where different fractions of martensite and austenite form depending on the peak temperature.

Zones 1−3 are the high-temperature regions where a significant fraction of non-equilibrium δ ferrite occurs [22].

In martensitic stainless steels, post-weld heat treatments (PWHT) are normally used to eliminate the δ ferrite, recover the mechanical properties, and reset the microstructure [24,25]. Typically, PWHT are restricted to only tempering operations in which the welds are tempered between 450–750 °C for times ranging between 30 min to 2 h [18,26]. Selection of the ideal tempering cycle is critical to achieve a balance between hardness and toughness and to avoid any drastic decreases in toughness due to embrittlement. For example, a 157 J difference in toughness has been documented in two samples subjected to two different heat treatment cycles. The toughness of the weld metal after being subjected to 650 °C − 2 h + 600 °C − 4 h was 170 J, as opposed to 13 J from a weld metal subjected to a tempering cycle of 675 °C and 615 °C. This difference in the toughness was attributed to the diffusion of Ni and C to the retained austenite during tempering at 675 °C, thus stabilizing the austenite films. Upon cooling down below room temperature (RT), the austenite films transform to fresh martensite, leading to a significant drop in toughness [27]. In addition, a systematic study has shown a sharp drop in toughness while tempering the steel between 500 and 650 °C, which was attributed the segregation of phosphorus, and other tramp elements, to the grain boundaries and thus embrittling the steel [28]. Chakraborty et al. also documented a similar observation where the toughness dropped from 60 J to 20 J while tempering at a range of 450–550 °C [29]. This drop, however, was attributed to the formation of Fe_2_C carbides in the martensite laths. Additionally, a further increase in tempering temperature led to the dissolution of the Fe_2_C phase and the toughness recovered back to 40 J.

Mirzaee et al. observed that, for 410 and 410 Ni steel, the ideal balance between toughness and hardness was observed when tempered at 450 °C and 550 °C. They attributed this to secondary hardening that resulted from primary and secondary carbide precipitation [30]. The toughness for both 450 °C and 550 °C tempered materials at a −18 °C test temperature were approximately 42 J and 38 J, respectively. Bilmes et al. also noted excellent toughness due to 600 °C PWHT of 13Cr–NiMo martensitic steel [31]. For the as-welded material, using single-stage tempering increased the toughness at the 20 °C test condition from 73 J to 116 J. While the majority of the work has focused on the tempering studies of welds, there are studies where austenitizing and tempering have been explored as well [30].

The above review of the literature clearly shows the efficacy of post-processing to effectively “reset” the microstructure and eliminate δ-ferrite. Although the extant literature provides a background towards understanding the science of processing 410 via additive manufacturing, there are significant gaps that need to be addressed:

1. The rapid cooling rates and complex thermal gyrations can lead to formation of non-equilibrium phases after solidification and from solid–solid phase transformation, as described previously. A detailed study on these complex phase transformations in 410 steel does not exist.
2. The role of such heterogeneity in microstructure on the mechanical properties is not well established.
3. As pointed out in the previous paragraph, there is no consensus in the additive manufacturing field on the PWHT treatments used to achieve a balance in properties of martensitic stainless-steel weld metal.

This study aims to address these gaps by performing systematic characterization of 410 SS samples that are fabricated using arc-based additive manufacturing. The associated scatter in mechanical properties was correlated to the microstructure, particularly the fraction of δ-ferrite. Following this, the effect of various heat treatment cycles—(i) austenitizing and (ii) austenitizing and tempering—to reset the microstructure and reduce the scatter in properties were evaluated. The results strongly suggest that the δ-ferrite can be linked to the reduction in toughness and the existence of local soft spots in the as-printed microstructure. A post-austenitizing heat treatment on the builds simply reset the microstructure by eliminating δ-ferrite, which led to a significant reduction in the scatter of tensile properties while simultaneously improving the impact toughness.

## 2. Materials and Methods

### 2.1. Control Strategy and Process Conditions During Sample Fabrication Process

All builds were manufactured using a Lincoln Electric wire-arc additive cell that consists of a six-degrees-of-freedom (DOF) ABB IRB-2600 robot with a Lincoln Electric PowerWave R500 power source. The wire used was a Lincoln Electric Blue Max MIG 410 (ER410) (Lincoln Electric Inc., Cleveland, OH, USA) with a 0.045-inch diameter. Sample fabrication included two vertical wall designs: one for tensile test specimens and the other for Charpy impact test specimens, based on ASTM standards E8/E8M and ASTM E23-09, respectively [32,33]. The dimensions for the Charpy test specimen wall were 362 mm (L) × 305 mm (T) × 12.8 mm (W), and the dimensions for the tensile test specimen wall were 600 mm (L) × 327 mm (T) × 12.8 mm (W). The wall for tensile samples was cut in half; one half was used in the “as-printed” state and the other half was subjected to heat treatment. All builds were constructed on one-inch-thick low-carbon steel build plates (ASTM A108). Each layer of deposited material was approximately 3.2 mm high and the total number of layers was 95 layers and 102 layers for the Charpy and tensile walls, respectively. Center-to-center bead spacing was 3.2 mm, such that each layer was built using a continuous print pattern measuring four beads in width. The welding parameters used for all builds utilized Lincoln Power Mode™ (Lincoln Electric Inc., Cleveland, OH, USA) with the following input settings: 2.5 kW welder power, a travel speed of 406 mm/min, and a wire feed speed of 5.6 m/min. Power Mode™ attempts to maintain a constant power input; therefore, the typical realized current and voltage values were around 15.5 V and 160 A. The shield gas was trimix (90% He/7.5% Ar/2.5% CO_2_).

### 2.2. Heat Treatment Schemes for Sample Fabrication

As mentioned in the previous section, two heat treatment cycles were used to develop post-processing protocols: austenitization heat treatment and tempering. Austenitization heat treatment was performed at 1050 °C for 60 min followed by air cooling. The austenitization temperature was determined based on the published literature which showed significant increase of average grain size above austenitization temperature of 1050 °C [34]. The heat treatments were done in an inert atmosphere to avoid any oxidation on the samples. After austenitization, one section of the block was retained to investigate the mechanical properties in the as-austenitized condition while the other sections were tempered. After austenitizing, the samples were tempered at 350 °C, 450 °C, 550 °C, 650 °C, and 750 °C for 90 min each, followed by air cooling. Tempering was also performed in an inert atmosphere. The heating rate used was 10 °C/min and thermocouples attached to the printed blocks were used to monitor the temperature during heat treatment. This approach yielded three different conditions: as printed, austenitized, and tempered samples.

### 2.3. Mechanical Testing

**Figure 1:**
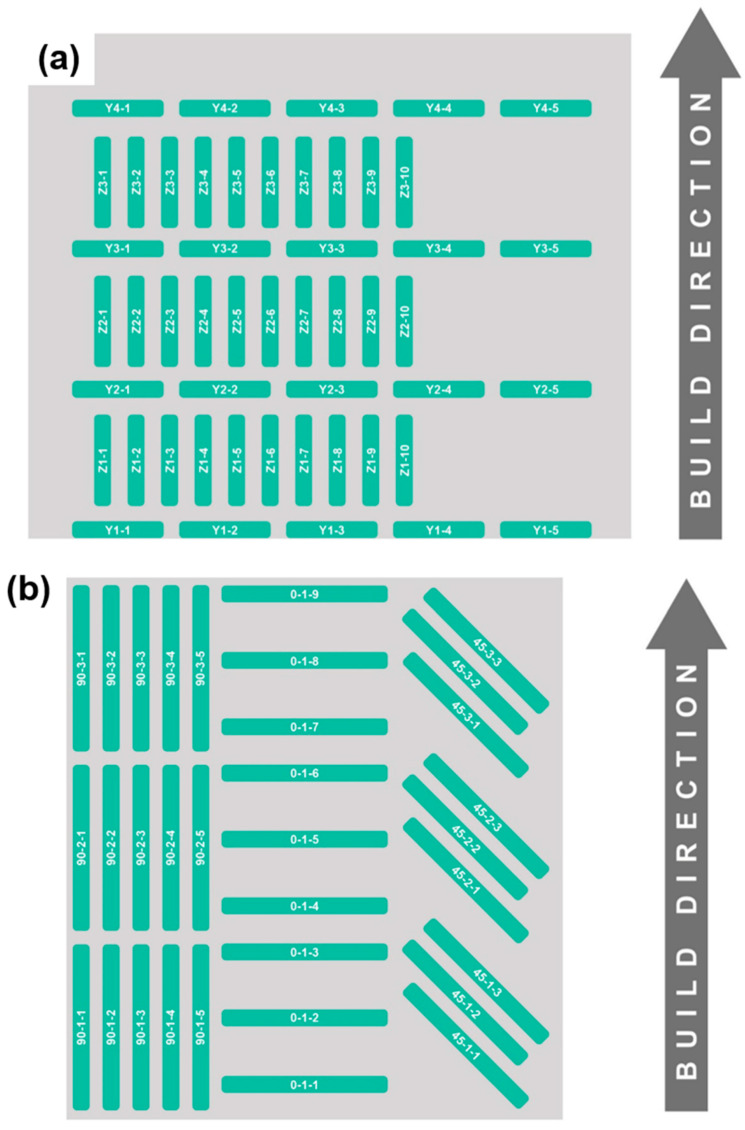
Pictorial representation of the (**a**) Charpy V-notch samples (305 mm tall × 362 mm long wall) and (**b**) tensile test samples (327 mm tall × 300 mm long wall-representative of the as-printed half).

Printed and heat-treated walls were machined for a set of samples for systematically preplanned tests, i.e., tensile tests, Charpy V-notch toughness test, hardness, and metallography. Figure 1 shows the layout drawing for sample locations within the builds for (a) Charpy toughness and (b) uniaxial tensile tests.

For Charpy V-notch tests, standard size samples (according to ASTM E23-09) with 55 mm long and 10 mm × 10 mm cross-section were machined from the fabricated wall. The samples were machined from two orientations, i.e., along the axis parallel to build direction *Z* and perpendicular to build direction *Y*. The *Y*-labeled samples had the notch along the *Z* (build) direction and vice versa. Three sets of samples parallel to the build direction *Z* were prepared at different heights of the wall, and four sets of samples perpendicular to the build direction *Y* were machined for toughness testing. In total, 20 *Y*-labeled and 30 *Z*-labeled Charpy samples were prepared (refer Figure 1a) to capture detailed toughness characteristics of the samples as a function of location and orientation against build direction. The tests were conducted on a Tinius Olsen Charpy Impact Machine (Tinius Olsen TMC, Horsham, PA, USA) with 407 J capacity at 0 °C to 200 °C test temperature with a step interval of 50 °C.

For the tensile test, sub-sized specimens based on ASTM E8/E8M were prepared. The specimens had overall length of 100 mm with gauge length and width 25 mm and 6 mm, respectively. The width of the grip section was 10 mm. In total, 33 samples were cut from three different orientations (refer Figure 1b), i.e., parallel, 45°, and perpendicular to the build direction. Specimens in those three orientations were machined at different heights from 5 mm above bottom zone of the built wall to 5 mm below the top surface. The tensile tests were carried out on a 100 kN capacity MTS SHM-07, four-pillar tensile frame at room temperature using a strain rate of 0.076 s^−1^. The yield strength was measured using a 0.2% offset method applied on tensile test data.

### 2.4. Microstructural Characterization

Samples for microstructural observation were machined from the center of build, 50 mm from the top, and 50 mm from the substrate region along the build direction and across wall thickness. The samples were mounted in conductive epoxy and later prepared using conventional metallographic procedures with 1 µm diamond paste finish. Two-dimensional hardness distribution measurement was conducted using a Leco LM100AT micro hardness tester (LECO Corporation, St. Joseph, MI, USA) using a 300 g load. Approximately 8 mm × 10 mm cross-sectional samples were sectioned from the *X*–*Z* plane of fabricated walls and hardness values were captured from above 2200 locations to formulate the hardness maps. The samples were then etched using with an etchant consisting of 80% H_2_O, 15% HCl, and 5% HNO_3_. Optical microscopy was performed using a Leica DMi8C optical microscope (Leica Microsystems Inc., Buffalo Grove, IL, USA). Scanning electron microscopy was performed using a JEOL 6500 FEG SEM (Tokyo, Japan) at an accelerating voltage of 20 kV with a probe current of 4nA. Electron back scatter diffraction was also acquired on the same equipment using an accelerating voltage of 20 kV and a probe current of 4nA with a step size of 1 μm. The analysis was performed on the EDAX TSL OIM analysis software v8.

## 3. Results Discussions

### 3.1. Characterization of the As-Printed Samples—Mechanical Testing

#### 3.1.1. Tensile Testing

**Figure 2:**
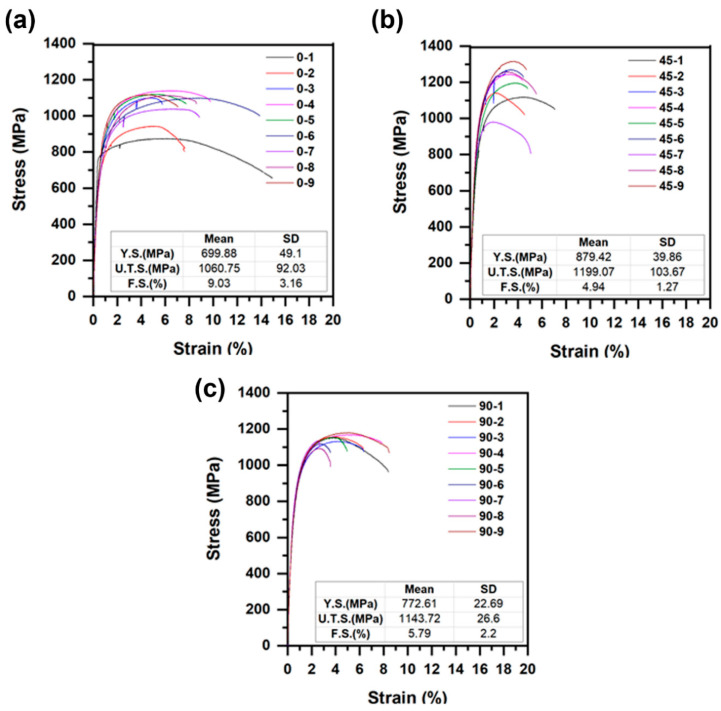
Stress versus strain curves: (**a**) perpendicular; (**b**) 45°; and (**c**) parallel to build direction, in as-printed samples. (Here, Y.S., U.T.S., and F.S. denote yield strength, ultimate tensile strength, and fracture strain, respectively.)

Proper verification of the build material strength is required before deployment of AM technology into the field. Figure 2a–c shows the tensile test data from nine representative as-printed samples, taken from the 0°, 45°, and 90° orientations. Specifically, in the samples extracted perpendicular to the build the direction, it was observed that the sample (sample 0–1) with lowest strength and highest ductility was extracted at locations close to the substrate (low-C steel). The dilution with low-C steel (base plate) resulted in a higher elongation and lower strength. For the as-printed samples, the average yield stress was 784 ± 84 MPa and the ultimate tensile stress was 1135 ± 97 MPa. The standard deviations of both yield stress and ultimate tensile stress were significantly high, which indicates extensive scatter in the tensile test data. The average fracture strain for the 0°-orientation samples was higher compared to the samples from the other two orientations. The standard deviation of the fracture strain values was high in all three directions. Average fracture strain for all as-printed samples was 6.5 ± 3%. The primary source of scatter can be attributed to alternating bands of tempered and untempered martensite, which form during continuous thermal cycling in the printed wall.

#### 3.1.2. CVN Toughness Testing

**Figure 3:**
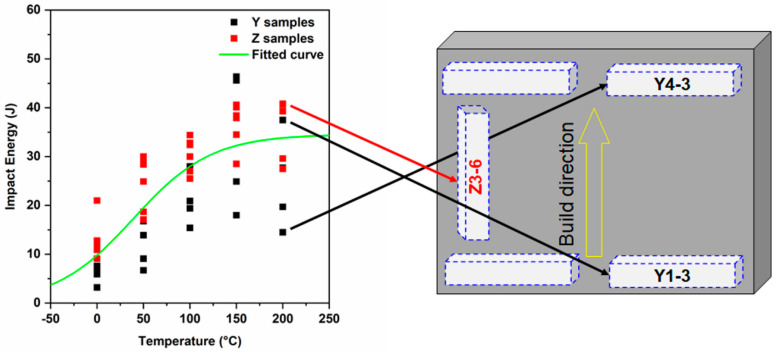
Charpy impact toughness results of perpendicular (*Y*) and parallel (*Z*) to build direction.

The results from the Charpy tests are shown in Figure 3, which also represents the anisotropy feature of the as-printed samples. The samples taken parallel to the build direction are labeled as *Z* samples and the samples taken perpendicular to the build direction are referred to as *Y* samples (Figure 1). Figure 3 shows the Charpy test data from 50 different samples machined from two different orientations and tested between 0 °C and 200 °C.

To measure the DBTT, the toughness values were fitted using a nonlinear regression curve fit based on the

(1) $$\mathrm{Impact}\;\mathrm{Toughness}=+\;B_{0}\tan h\left(\frac{T-T_{0}}{C_{0}}\right)$$

where A_0_, B_0_, C_0_, and T_0_ are constants and T is the temperature. The DBTT is defined as the temperature corresponding to half the height of the transition curve. The ductile-to-brittle transition temperature, determined using this approach, was ~70 °C. The upper shelf energy was approximately 35 J and lower shelf energy was below 5 J. Overall, the samples extracted along the *Y* direction showed lower toughness (for example, at ~75 mm above base plate, the toughness of the *Y* sample was around 7 J, but for the *Z* sample, the toughness was approximately 17 J at 0 °C) compared to samples extracted along the *Z*-axis, indicating directionality in the printed samples. While the samples extracted along the *Z*-axis consistently showed higher toughness values than the samples from the *Y* direction, there was also a significant scatter within the data. For example, the sample Y1-3 tested at 200 °C showed a toughness of 37 J while another sample Y4-3 showed a toughness of 14 J, indicating that in addition to the direction, the toughness is also a function of the notch microstructure. Note that the scatter band also starts to widen at higher temperatures, showing a heterogeneity in the microstructure. In addition, samples obtained from the bottom region of the build exhibited higher toughness values than those from the top. This trend occurred at all temperatures, especially in samples extracted along the *Y* direction where this was most pronounced.

**Figure 4:**
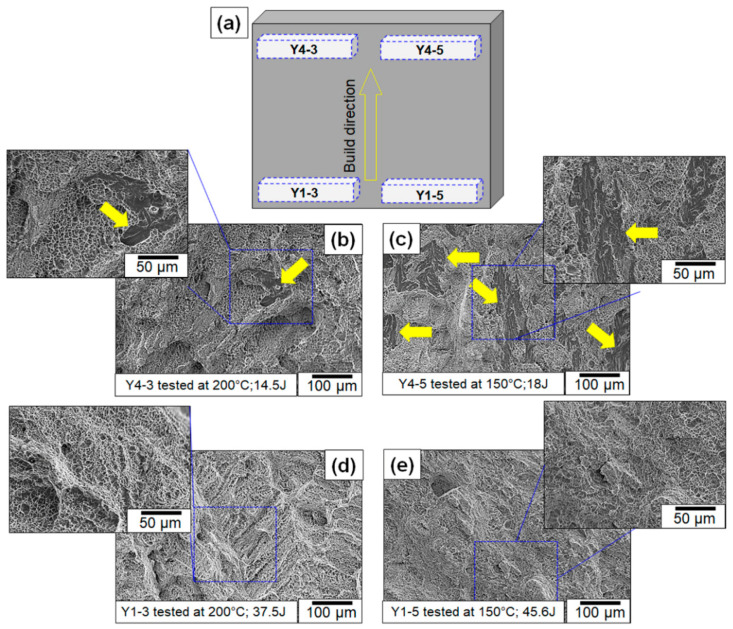
(**a**) Pictorial diagram showing the position of Charpy samples on the fabricated wall. Fractographs of Charpy samples sectioned from (**b**,**c**) top and (**d**,**e**) bottom of the build. Yellow arrows are showing brittle failure modes in the above figures.

Fractography was performed to investigate the differences in surface morphology among the various samples and the results are summarized in Figure 4. Figure 4a shows the pictorial diagram of analyzed Charpy samples on the fabricated wall. The fractography of samples extracted from the bottom-most region of the build (where the build meets the base plate) showed no brittle failure (Figure 4d,e), but samples extracted at 275 mm away from the base plate displayed brittle failure (Figure 4b,c showing cleavage fracture with a flat surface as pointed by yellow arrows). The point count method was used to estimate the fraction of brittle failure zones in the images, which ranged from 1.96% to 18.9%. The fractography also corroborates the toughness data because the samples extracted from the top of the plate (Y4 samples) had a higher fraction of brittle failure.

**Figure 5:**
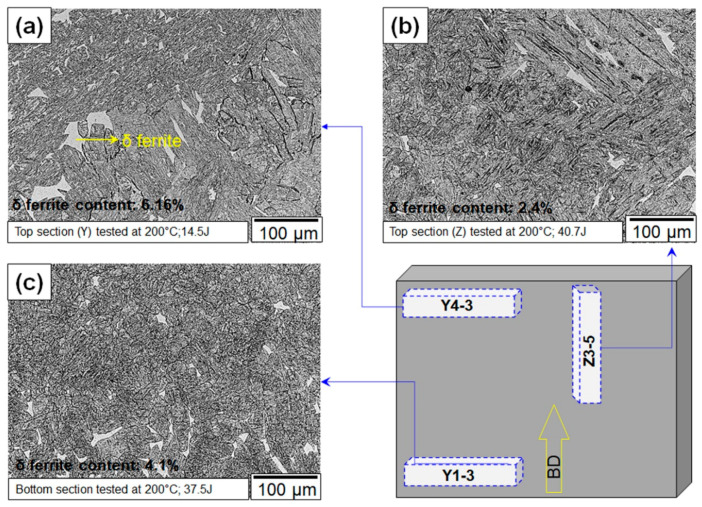
Representative microstructural observation on failed Charpy samples sectioned (**a**,**c**) perpendicular and (**b**) parallel to build direction from different build heights.

In addition, the failure morphology also suggested that the regions where shear propagation occurred could also correspond to the locations where δ-ferrite existed. Figure 5 illustrates optical micrographs showing the representative microstructure taken from different locations in as-printed wall. The areas with a bright contrast and non-characteristic relief correspond to δ ferrite. The area fraction of δ ferrite was measured at each location using the micrographs and some representative images are showed below. The results indicated that the top portion exhibited a greater amount of δ phase than the other locations. This suggests that for *Y*-labeled samples especially, the top region had comparatively higher δ-ferrite contributing to the lower toughness values. Therefore, hardness testing at various locations in the builds and optical microscopy were performed.

#### 3.1.3. Hardness Mapping

**Figure 6:**
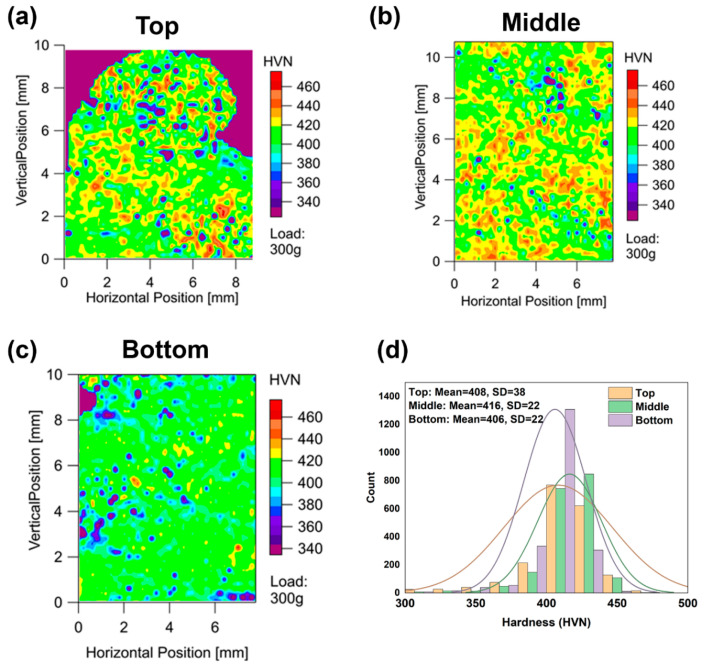
Hardness maps from different locations in the build height: (**a**) top; (**b**) middle; (**c**) bottom of the build. (**d**) Hardness distribution histogram of each region.

Hardness mapping was used to investigate the local heterogeneity of the microstructure and rationalize the toughness behavior. The hardness maps were performed on the top, middle, and bottom regions of the builds. Figure 6 shows the hardness maps from the (a) top, (b) middle, and (c) bottom regions of the as-printed samples. The contour maps clearly show that there are significant soft spots in the samples. The soft spots appeared sporadically and did not form a pattern. These soft spots can be attributed to formation of δ-ferrite during the wire arc additive manufacturing process. The hardness distribution histogram of each region was plotted and fitted using a Gaussian distribution (Figure 6d). The data shows a higher standard deviation in the sample from the top section of the build (38 HVN compared to 22 HVN in the middle and bottom), indicating a higher probability of scatter in the samples from those locations. Higher thermal cycling leads to homogenization of the microstructure and, therefore, the samples from the bottom should have a slightly lower scatter in properties.

#### 3.1.4. Microstructure Characterization

**Figure 7:**
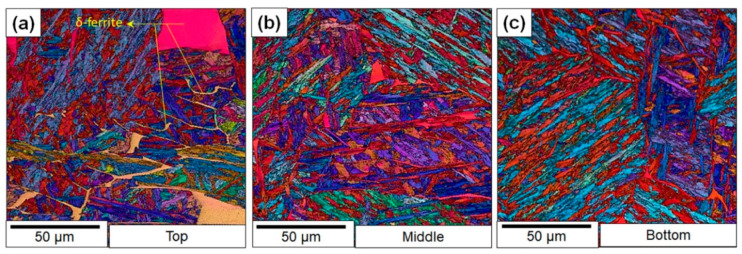
Electron backscatter diffraction (EBSD) micrographs of as-printed samples sectioned from different locations on the wall: (**a**) top; (**b**) middle; (**c**) bottom.

To confirm the results from the hardness tests and also rationalize the large scatter in toughness, a detailed characterization campaign was initiated. Figure 7 shows the electron backscatter diffraction (EBSD) micrographs of the samples from various locations of the builds. The data show that the bottom regions are predominantly martensitic with limited δ-ferrite stringers. The δ-ferrite stringers present are along the inter-dendritic regions with similar orientations, which suggests that the δ-ferrite formed during solidification and not during the thermal cycling. The progressive increase in the δ-ferrite in the samples extracted from the upper region of the build, which undergoes fewer thermal cycles, supports this interpretation. The morphology of the δ-ferrite changes from stringers to blocky δ-ferrite.

**Figure 8:**
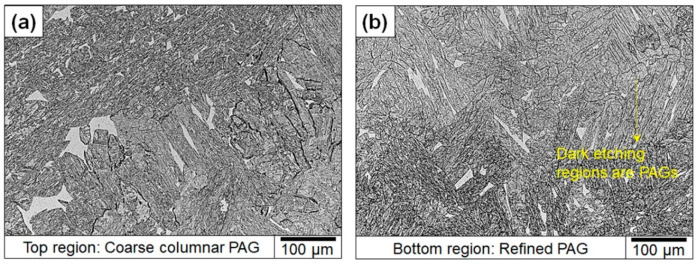
Optical micrograph showing differences in grain microstructure at (**a**) top and (**b**) bottom of the builds in as-printed condition.

It is possible that inclusions contributed to brittle failure by serving as crack nucleation points. This hypothesis was ruled out in the present case because the fractography analysis did not show any evidence of cracks nucleating from the inclusions. Therefore, to rationalize the cause for the toughness scatter, the notch microstructures of samples were studied. The corresponding microstructures are presented. The microstructure shows that the difference in δ-ferrite fractions between the *Y* and *Z* samples. The average percentage of the area that was covered by δ-ferrite in the *Y* samples was 5% ± 1%, whereas for *Z*-labeled samples, it was 3% ± 1%. In addition, the optical micrographs (Figure 8) also showed a more refined prior austenite grain size in the bottom regions of the builds compared to the top regions, where coarse columnar grains were observed (Figure 8a). Both the reduced δ-ferrite content and the refinement of the prior austenite grains could have also contributed to the increased toughness in the bottom locations of the build.

Based on the characterization and property evaluation above, two important events can be hypothetically highlighted in the microstructure evolution at the bottom region, as listed below:

1. Elimination of δ-ferrite during thermal cycling: The bottom locations undergo significantly more thermal cycling. As pointed out previously, there are five distinct zones in the reheated heat-affected zones [22]. Of the five zones, repeated thermal cycling in the fully austenitic zone would lead to the gradual dissolution of the δ-ferrite pockets [18]. Upon cooling, the reheated zone transforms from the fully austenitic region to martensite without any δ-ferrite, depending on heating and cooling rates. This results in the gradual decrease in δ-ferrite during every cycle in which the temperature is in the fully austenite region. This repeated reheating and cooling in the bottom regions of the build could have contributed to the reduction in δ-ferrite as shown in the micrographs and in the hardness results.
2. Grain refinement: During the reheating cycles, the material is reheated to the inter-critical temperature in which new austenite reversion occurs. The reverted austenite recrystallizes, leading to the refinement of the prior austenite grains [35]. The recrystallization of the austenite is driven by the high stored energy form dislocations which form via shear mechanism during the retransformation to austenite. Dislocation densities exceeding 10^11^/cm^3^ have been documented in reverted austenite [36]. This only occurs when the heating rate is beyond a critical value, typically ~100 °C/s. Upon cooling, these refined austenite grains transform to martensite with an improved toughness, due to a reduction in the prior austenite grain size. When the heating rates are much higher and close to 1000 °C/s, the refinement is dramatic due to a different mechanism. In this case, the refinement is dominated by a packet refinement mechanism, as opposed to prior austenite grain refinement. When the alloy is rapidly heated at the mentioned heating rates to accomplish austenite reversion, the reversion occurs without any recrystallization. The high dislocation density leads to more heterogeneous nucleation of martensite with a higher defect density. Due to this heterogeneous nucleation, martensite formation is suppressed via bivariant blocks or packets, and all of the 24 different variants appear randomly [35]. Thermal cycling therefore breaks down the columnar austenite grains that form during solidification by either recrystallization or packet refinement. The exact mechanism by which the refinement occurs is still unclear, and more work is necessary to develop a fundamental understanding of the microstructure evolution.

### 3.2. Experimental Results on the Heat-Treated Samples

Based on the characterization results of as-built material and the literature, the low toughness was attributed to presence of δ-ferrite. Therefore, heat treatments were designed to eliminate the δ-ferrite. To that effect, two different types of heat treatments were performed: (a) austenitization at 1050 °C for 60 min followed by water quenching and (b) austenitization and tempering at 350 °C, 450 °C, 550 °C, 650 °C, and 750 °C temperatures for 90 min each. The aim of the austenitization treatment was to understand if the low toughness was purely a function of the high δ-ferrite content in the builds or if it was also due to the presence of untempered martensite. Therefore, the austenitization heat treatment was designed to eliminate the δ-ferrite while having a homogenous untampered martensite.

#### 3.2.1. Tensile Testing on Austenitized Samples

**Figure 9:**
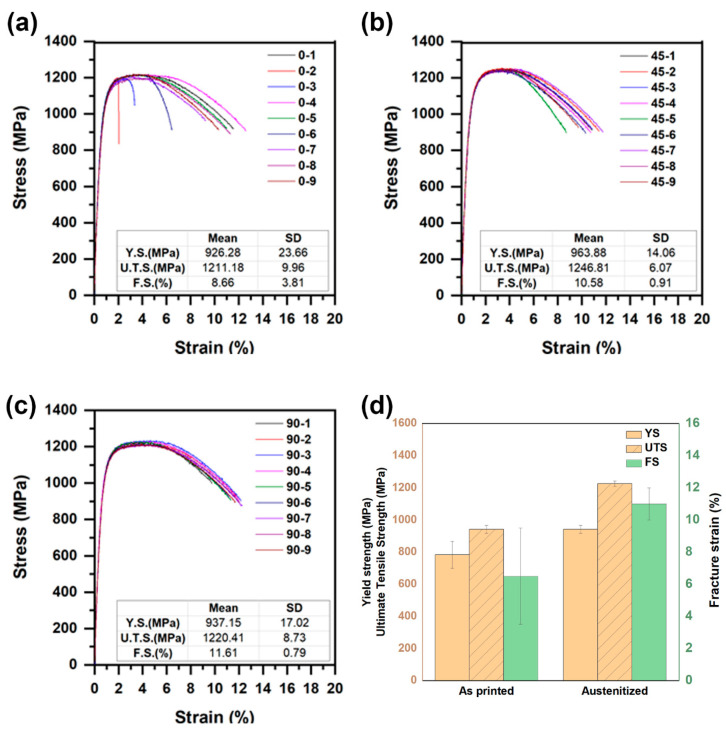
Stress versus strain curves (**a**) perpendicular; (**b**) 45°; (**c**) parallel to build direction for austenitized samples. (**d**) Comparative bar graph of yield stress, ultimate tensile stress, and fracture strain data in as-printed and austenitized samples. (Here, Y.S., U.T.S. and F.S. denote yield strength, ultimate tensile strength, and fracture strain, respectively.)

The results of tensile tests the on representative 27 samples sectioned in three different directions are presented in Figure 9a–c. The scatter of tensile properties disappeared after austenitization. Three austenitized samples (with 0° orientation) showed fracture with a very low strain percentage which may have been due to the presence of discontinuities in microstructure. The yield and ultimate stress levels were comparable in all three directions for the samples, showing there was no directionality in tensile stress behavior. Figure 9d shows an overall effect due to austenitization as compared to as-printed samples. The standard deviation among both yield (24 MPa) and ultimate (17 MPa) tensile strength was significantly lower compared to the as-printed cases. In particular, the standard deviation for yield strength reduced 84 MPa on as-printed samples to 24 MPa as depicted above; a 70% reduction in the scatter on tensile strength was possible due to austenitization treatment. Austenitizing helped to enhance the yield stress from 784 MPa to 942 MPa and ultimate tensile stress from 1135 to 1226 MPa. Excluding three 0°-orientation samples with very low fracture strains, the average fracture strain of the remaining austenitized samples was 11% ± 1%. This confirms an increase the in average fracture strain value with decreased scatter in the number of individual fracture strains. The drastic reduction in scatter after austenitizing treatments suggests that the scatter in the as-built material was mostly associated with microstructural heterogeneity, together with the dispersion of δ ferrite.

#### 3.2.2. CVN Toughness and Hardness Mapping on Testing on Austenitized Samples

**Figure 10:**
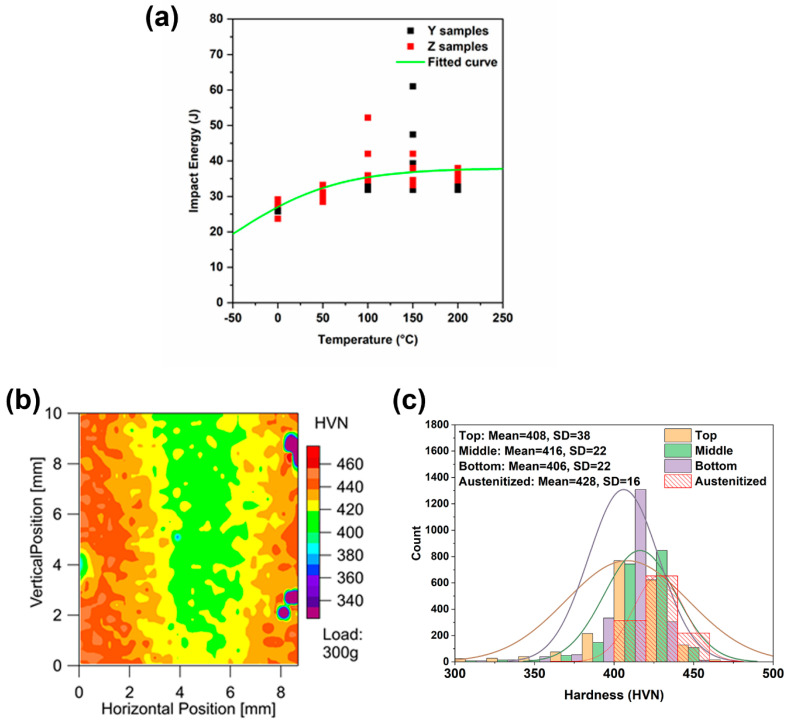
(**a**) Charpy impact toughness results on austenitized samples sectioned perpendicular *Y* and parallel *Z* to build direction, (**b**) representative hardness map of austenitized samples, and (**c**) comparative study showing distribution in hardness of austenitized samples against as-printed samples.

Figure 10a shows the Charpy test data from the austenitized samples. The average impact energy, even at 0 °C, was 27 J with a standard deviation of 2 J, in contrast to the average toughness of the as-printed samples at 50 °C, which was 22 J with a standard deviation of 8 J. The results clearly demonstrate the beneficial effect of eliminating δ-ferrite in the samples. The scatter and anisotropy in the samples were also significantly lower.

Hardness maps were also performed after austenitization (Figure 10b). The hardness map shows a gradual decrease in the hardness from the surface to the core. This is attributed to the fact that the surface cools faster than the core, leading to a finer martensitic microstructure on the surface and progressively decreasing hardness towards the middle of the sample. The histogram values of the hardness results are plotted in Figure 10c and are overlaid with a Gaussian fit of the distribution. The resulting distribution is compared with the distribution obtained in the as-printed samples (top, middle, and bottom regions). The results show a narrower distribution of hardness with a higher mean hardness (428 HVN) and a smaller standard deviation (16 HVN) compared to the top region of the as-printed samples (mean 408 HVN and standard deviation of 38 HVN). The increase in the hardness suggests the absence of δ-ferrite, and the decreased standard deviation suggests that the microstructure is homogenous.

#### 3.2.3. Microstructural Observations on Austenitized Samples

**Figure 11:**
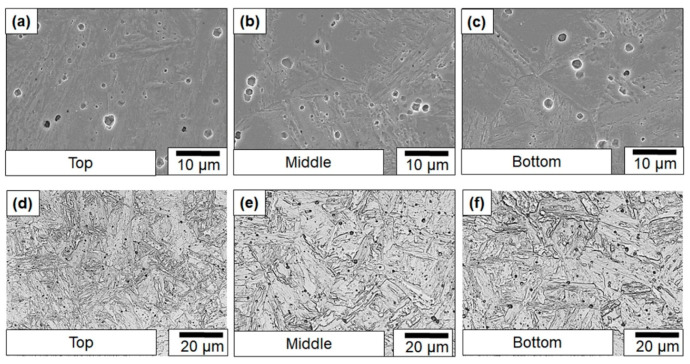
(**a**–**c**) Scanning electron and (**d**–**f**) optical micrographs of austenitized samples sectioned from the (**a**,**d**) top, (**b**,**e**) middle, and (**c**,**f**) bottom of the wall.

Microstructural observation was necessary to validate the hypothesis that was formed based on the tensile test data. Detailed microstructural observations, using SEM and optical imaging, are shown in Figure 11. The microstructure predominantly consisted of an untempered martensite. δ-ferrite was not observed in any of the images captured in three different locations of the build, indicating a successful elimination of δ-ferrite and complete reset of the microstructure. This also corroborated the hypothesis that the elimination of δ-ferrite will improve toughness even when the matrix microstructure is 100% untempered martensite. A similar observation was previously documented by Carrouge et al., where a martensitic microstructure containing 14% δ-ferrite had a DBTT 50 °C (DBTT of −46 °C) higher than a 100% martensitic microstructure (DBTT of −98 °C) [22]. They also observed that, by reducing the δ-ferrite from 14% to 2%, it was possible to recover the DBTT to a value close to the parent material. The DBTT of the microstructure with the 2% δ-ferrite was ~−87 °C. This is consistent with the idea that complete elimination of δ-ferrite leads to a drastic improvement in toughness of the 410 stainless steel samples fabricated via a wire-arc-based additive manufacturing.

While austenitizing the samples improved the toughness without a loss in hardness, tempering is typically required to further improve the toughness. Tempering also sometimes can help in slightly enhancing hardness, due to the precipitation of alloy carbides [30]. However, the tempering curves for additively manufactured 410 steel are not available and, as pointed out in the literature review section, there is significant uncertainty in the literature regarding the post-tempering embrittlement of martensitic steels. Therefore, a detailed study of the effects of tempering on the hardness and toughness was performed.

#### 3.2.4. Microstructural Observation on the Tempered Samples

**Figure 12:**
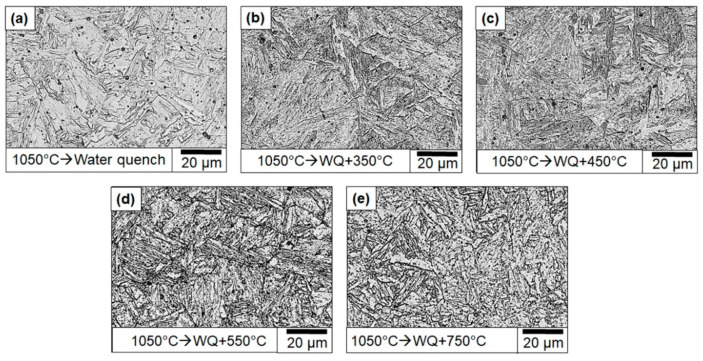
Optical micrographs of (**a**) austenitized followed by water-quenched and tempered samples with tempering temperature; (**b**) 350 °C; (**c**) 450 °C; (**d**) 550 °C; (**e**) 750 °C.

Since alloy carbides can form in the early stages of tempering in 410 steels, it is reasonable to expect a certain degree of secondary hardening [30,37]. To expect further improvement of the toughness, the samples were tempered at temperatures between 350 °C to 750 °C in increments of 100 °C. Figure 12 shows the optical microstructure of the as-austenitized samples compared with the selected tempered samples. The optical micrographs clearly show that extensive tempering occurs only at temperatures beyond 550 °C.

**Figure 13:**
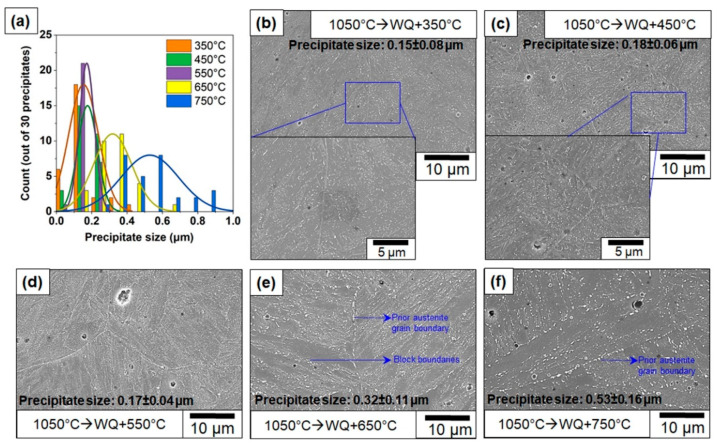
(**a**) Histogram of precipitate counts in different temperature tempered samples and scanning electron micrographs showing precipitates in samples with tempering temperature; (**b**) 350 °C; (**c**) 450 °C; (**d**) 550 °C; (**e**) 650 °C; (**f**) 750 °C.

To investigate this in more detail, SEM was performed, and the results are shown in Figure 13. Figure 13a shows a graphical representation of increase in precipitate sizes with increase in tempering temperature. The average precipitate size increased significantly when the tempering temperature was 550 °C. While apparent microstructural changes after tempering at 350 °C have not been identified in an optical microscope, the SEM images clearly show the presence of carbide precipitates in the samples tempered at 350 °C. The insets in Figure 13b,c show higher magnification of the microstructure, clearly demonstrating the presence of carbides in the microstructure. The carbide distribution is homogenous and needle-shaped. Discerning the nature of these carbides using advanced microscopy is a subject of further research and is beyond the scope of this manuscript. The presence of these needle-shaped carbides has been documented in a previous study [30]. The authors identified these carbides as Fe_2_C and associated the formation of Fe_2_C with a drop in toughness, although the exact mechanism of toughness reduction has not been determined. With increasing temperatures, the carbides coarsen and form lenticular or globular carbides, typically M_23_C_6_. While the size distribution of the carbides at tempering between 350–550 °C is homogenous, at temperatures beyond 550 °C, a bimodal distribution of carbides occurs (Figure 13e,f). The carbides near the prior austenite grain boundary are coarser compared to the carbides in the block boundaries.

**Figure 14:**
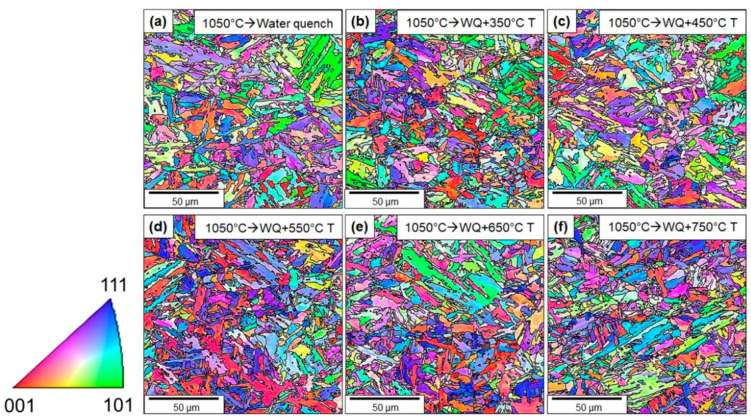
EBSD micrographs of (**a**) austenitized followed by water-quenched and tempered samples with tempering temperature; (**b**) 350 °C; (**c**) 450 °C; (**d**) 550 °C; (**e**) 650 °C; (**f**) 750 °C.

EBSD inverse pole figure (IPF) color maps of austenitized and tempered samples are shown in Figure 14, which also illustrates the grain or packet boundaries with a misorientation angle ≥15°. The martensite block sizes from EBSD micrographs were also measured using the ImageJ software (NIH, Maryland, USA) and are presented in Table 1. It can be observed that increasing the tempering temperature above 550 °C resulted in an increase in the martensite block size, while tempering between 350–550 °C did not contribute to the coarsening of the martensite blocks. 

Hardness testing and Charpy toughness testing were performed to determine which tempering treatment achieves the optimum toughness and hardness for tooling fabricated with 410, and the results are discussed in the next section.

#### 3.2.5. CVN Toughness and Hardness Measurements on Tempered Samples

**Figure 15:**
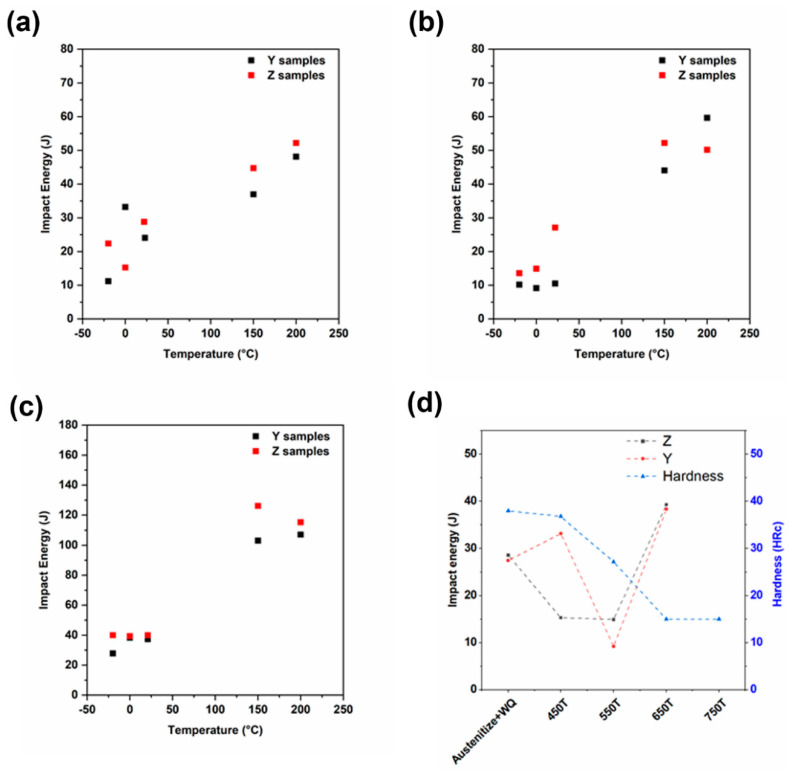
Charpy impact toughness results on samples tempered at (**a**) 450 °C; (**b**) 550 °C; (**c**) 650 °C. (**d**) Plot showing toughness and hardness of different samples at 0°C test temperature.

Figure 15a–c shows the results of the Charpy V-notch toughness test that was performed on tempered samples at different temperatures. The samples from three different tempering temperatures (450 °C, 550 °C, and 650 °C) were selected for Charpy V-notch tests. Toughness measurements were performed on samples tempered at these temperatures because it has been reported that a significant drop in toughness occurs when tempering between 400–510 °C [30]. Therefore, temperatures in the middle of that range, just outside the range, and well above the range were selected for further investigations. The samples were tested at −20 °C, 0 °C, 22 °C, 150 °C, and 200 °C. The changes in the impact toughness at 0 °C are plotted in Figure 15d, as a function of the different processing treatments in both the *Z* and *Y* directions. In addition, the right-hand axis shows the changes in the Rockwell hardness as a function of the tempering treatment. The data shows a drastic drop in toughness during tempering between the 450–550 °C in the *Z* direction. However, the toughness when testing along the *Y* direction showed a similar drop only for tempering at 550 °C. While the maximum toughness at 0 °C was obtained after austenitizing and tempering at 650 °C, the hardness values dropped significantly (~15 HRC). The best-case scenario where the optimum balance between the hardness and toughness can be obtained is austenitizing and tempering at 450 °C. The reason for the anomalous drop in the toughness, specifically for the *Z*-axis samples, is not understood and is a subject for further research.

This work has attempted to set the stage towards documenting, understanding, and mitigating the challenges of arc-based additive manufacturing of martensitic stainless steels. Mirzaee et al. observed that the toughness of 450 °C- and 550 °C-tempered wrought 410 steel samples is around 13 J and 21 J, respectively, at a −18 °C test temperature [30]. Metal big-area additively manufactured *Z*-directional samples tempered at 450 °C and 550 °C showed toughness levels of 22 J and 14 J, respectively, at the −20 °C test condition.

## 4. Conclusions

This study systematically documented the scatter in mechanical properties and correlated it to the microstructural heterogeneity in a SS 410 sample fabricated using arc-based additive manufacturing. A solution to mitigate the scatter in those mechanical properties was also presented here.

- The as-fabricated samples showed significant scatter in their properties. The average yield strengths of the as-fabricated condition in 0°, 45°, and 90° oriented samples were 700 ± 49, 879 ± 40, and 773 ± 23 MPa, respectively. In addition, there was significant scatter in the toughness values because the samples extracted from the bottom of the build had a higher toughness compared to those present in the top. This has been attributed to the formation of δ-ferrite in the top zone of the build and the smaller amounts of the δ-ferrite in the bottom regions of the build.
- To eliminate the scatter in properties, post-processing heat treatments were employed. Austenitization was performed at 1050 °C to eliminate the δ-ferrite in the builds. Samples after austenitization showed a significant reduction in the scatter of the toughness and tensile data. Complimentary microscopy and hardness testing indicated that this increase in toughness and reduction in scatter can be attributed to the elimination of δ-ferrite and the homogenization of the microstructure.
- Tempering studies were also conducted on the austenitized specimens, at different temperatures between 350–750 °C, in increments of 100 °C. The studies showed a reduction of toughness while tempering in the 450–550 °C range. It was also observed that the decrease in toughness was more pronounced in the samples along the *Z* direction (build direction) compared to the *Y* direction. This is a subject for future research.

Overall, this study has documented the challenges associated with the scatter in toughness. In addition, it also outlines the opportunities for alloy development efforts within the 410 space. For instance, this study has conclusively shown that the scatter in 410 is primarily driven by the formation of δ-ferrite. Therefore, the alloys used for additive manufacturing should balance alloy composition and deposition parameters to effectively allow primary δ-ferrite to completely transform to austenite during solidification. Of the commercially available martensitic stainless-steel wires, 420 [38] and 410–NiMo [25] can be used to reduce the δ ferrite formation. In addition, novel compositions can also be designed, using CALPHAD approaches, to create compositions tailored for additive manufacturing.

## Figures and Tables

**Table 1:** Effect of tempering temperature on martensite block size (Here ‘WQ’ refers to Water Quench and ‘T’ refers to tempering temperature).

	1050 °C + WQ	350 T	450 T	550 T	650 T	750 T
Avg. block width (µm)	1.4 ± 0.4	1.7 ± 0.5	1.7 ± 0.5	1.7 ± 0.5	2.2 ± 0.5	2.3 ± 0.5
